# Muscle power, contraction velocity and functional performance after stroke

**DOI:** 10.1002/brb3.1243

**Published:** 2019-02-28

**Authors:** Joanna Kostka, Marta Niwald, Agnieszka Guligowska, Tomasz Kostka, Elżbieta Miller

**Affiliations:** ^1^ Department of Physical Medicine Medical University of Lodz Lodz Poland; ^2^ Department of Geriatrics, Healthy Ageing Research Centre Medical University of Lodz Lodz Poland

**Keywords:** functional performance and age, functional performance and stroke, knee extensor muscles, muscle contraction velocity, muscle function, power measurement

## Abstract

**Objective:**

The goal of this study was to describe muscle function deficit in patients after stroke as well as to define the relationship between maximal muscle power (P_max_) and optimal shortening velocity (υ_opt_) with functional efficiency in stroke survivors.

**Material and Methods:**

A total of 134 participants were enrolled in the study, including 67 patients after a stroke and 67 volunteers, matched for age and sex (controls). Functional performance was measured with the timed Up and Go test (TUG) and additionally with Rivermead Motor Assessment (RMA) and Barthel Index (BI) in stroke survivors. To assess P_max_ and υ_opt_ of the knee extensor muscles, a specially equipped Monark cycle ergometer was used.

**Results:**

The power generated by stroke survivors was 49.6% that of their peers and muscle contraction velocity was 65.5%. P_max_/kg and υ_opt_ were associated with TUG outcomes in both groups. P_max_/kg and υ_opt_ were associated with age in the control group, but not in patients after stroke. In multivariate analysis in patients after stroke, TUG was better predicted by P_max_/kg or υ_opt_ than by the age. In stroke survivors, both P_max_/kg and υ_opt_ were related to the BI and to the RMA total results. Both BI and RMA total were not determined by age.

**Conclusions:**

Muscle power and muscle contraction velocity in patients who have had a stroke within three months have reduced markedly. These factors significantly affect functional performance. Muscle power and optimal shortening velocity are more important determinants of functional status than age in these stroke survivors.

## 1 INTRODUCTION

Stroke is one of the leading causes of disability in older adults. One of the reasons for this is a deficit in muscle function after stroke. From the point of view of the functional performance, maintenance of lower limb muscle mass and function seems to be crucial (Guralnik, Ferrucci, Simonsick, Salive, & Wallace, 1995). The efficiency of the lower limbs is largely responsible for the ability to function independently, because it allows one to stand up, maintain balance and control, and, most of all, ensure an efficient gait that is crucial for daily activities.

Muscle function can be considered in several aspects: muscle strength, muscle power and muscle contraction velocity. These parameters may in different ways determine the functional performance of patients. Muscle strength and its role in everyday functioning in patients after stroke are most often described in the literature (Bohannon, 2007; Kostka, Czernicki, Pruszyńska, & Miller, 2017). Meanwhile, some studies suggest that muscle power and muscle contraction velocity may be more important factors affecting functional efficiency than muscle strength, especially in the elderly (Bean et al., 2003; Clémençon, Hautier, Rahmani, Cornu, & Bonnefoy, 2008; Kostka, Czernicki, & Kostka, 2014). Some important activities of daily living require not only muscle strength (the ability to generate force), but also adequate power, which means the ability to generate force in a sufficiently short time (power is a product of force and velocity, Stavric & McNair, 2012).

Studies by some authors indicate a deficiency in muscle strength in patients after stroke both in the affected and unaffected side (Gerrits et al., 2009; Prado‐Medeiros et al., 2012). In the limited research on deficits of muscle power after stroke, the authors compare the power deficit in relation to the unaffected side (Bohannon, 1992; Dawes et al., 2005) which does not entirely reflect the real deficit. With the exception of our preliminary study (Kostka, Fajkowska, & Miller, 2017), in the available literature we have found only one study in which the deficit of maximal muscle power in stroke survivors was compared with healthy peers (Stavric & McNair, 2012) and have not found any study on optimal muscle contraction velocity.

The goal of this study was to describe muscle function deficit in relation to peers who had not suffered a stroke. We also wanted to define the relationship between maximal muscle power (P_max_) and optimal shortening velocity (υ_opt_) with functional efficiency in stroke survivors in a relatively short period after a cerebrovascular incident.

## 2 MATERIALS AND METHODS

### 2.1 Subjects

A total of 134 participants (44 women and 90 men) were enrolled in the study, including 67 patients (22 women and 45 men) who had been hospitalized after a stroke in the Neurological Rehabilitation Department of Dr. K. Jonscher Municipal Medical Centre in Lodz (patients) and 67 volunteers, matched for age and sex with group of patients, who did not suffer a stroke and were consecutively recruited during programs coordinated by the Geriatric Department of the Medical University of Lodz (controls). Inclusion criteria for post stroke survivors were as follows: time after stroke between 2 weeks and 3 months, stable clinical condition, unilateral paresis of a leg, ability to understand and execute commands, ability to perform exercise tests, preserved walking ability (supportive equipment was allowed) and informed written consent to participate in the study. Patients with severe spasticity (>2 Ashworth scale), significant limitation of range of motion in lower limbs, severe cognitive deficits or any contraindications to exercise testing were excluded from this study.

The study was approved by the Bioethics Committee of the Medical University of Lodz.

### 2.2 Protocol

All the participants underwent physical examination before the study. During the interview, information on socioeconomic status and current and previous illnesses was obtained. Anthropometric measurements (body mass and height) were also taken from all the participants and based on those measurements, body mass index (BMI) was calculated (kg/m^2^).

### 2.3 Functional performance

Functional performance was measured with the timed Up and Go test (TUG) in both groups. Additionally, a functional measurement specific for stroke patients ‐ Rivermead Motor Assessment (RMA) and Barthel Index (BI) were completed for stroke survivors and used for further analyses (in the control group the "ceiling effect" was achieved in these tests). TUG (Flansbjer, Holmbäck, Downham, Patten, & Lexell, 2005; Podsiadlo & Richardson, 1991) assesses basic functions of daily life: standing up from a standard height chair, walking a short distance (3 m), returning and sitting back down. Time was measured on a stopwatch to the nearest 0.1 s. RMA (Collen, Wade, & Bradshaw, 1990; Lincoln & Leadbitter, 1979) is a widely used scale of motor function in patients after stroke. The scale consists of three sections: gross function, leg and trunk, and arm. A total score for each item ranges from 0 to 13 for RMA gross function, from 0 to 10 for RMA leg and trunk, and from 0 to 15 for RMA arm. The RMA total score was used for further analysis. BI (Collin, Wade, Davies, & Horne, 1988) is a 10‐item instrument, used also in post stroke patients assessment (Wu, Wang, Teng, Huang, & Shang, 2015), measuring level of functional independence in activities of daily living. The total score ranges from 0 (minimum independence) to 20 (maximum independence).

### 2.4 Muscle maximum power (P_max_) and optimal shortening velocity (υ_opt_) measurements

To assess P_max_ and υ_opt_ of the knee extensor muscles, a specially equipped Monark cycle ergometer was used as previously described (Kostka, 2005). Pedaling velocity (υ), force (F) and power output (P) were calculated each 5 ms and then averaged over each downstroke period. Before the measurement, a 5‐min warm‐up was done by each patient. Next, the participants were asked to perform two 8 s sprints (with at least a 5‐min rest between the two tests) with friction loads of 0.25 N/kg and 0.35 N/kg of body mass or less (0, 1–0, 2 N/kg) in cases where the patient had difficulty with initiating the pedaling at a given load. During the test, patients were encouraged to ride as quickly as possible. The highest value of P (P_max_) and optimal shortening velocity (υ_opt_ – velocity at which the power reaches a maximum value) were calculated from a third‐order polynomial function. P_max_ was expressed in relation to body mass [P_max_/kg (W/kg)]. υ_opt_ was given in number of rotations per minute (rot/min).

### 2.5 Statistical analysis

Data were verified for normality of distribution and equality of variances. To compare the results between the groups, a one‐way analysis of variance (ANOVA), Mann‐Whitney test, and chi‐square test with Yates’ correction for 2 × 2 tables were used. The deficits of P_max_, υ_opt_ and TUG for patient after stroke were given as a percentage of the results obtained by the control group. To identify the quantitative variables influencing muscle power and the shortening velocity, the Pearson or Spearman correlation coefficients were used. To identify the most important determinants of functional tests, the multivariable analysis with forward selection option was made. Prior to this analysis, the data in which the distribution was not consistent with the normal data were transformed logarithmically. Data were presented as mean (±*SD*). The level of statistical significance was set at *p* < 0.05.

## 3 RESULTS

Baseline characteristics of the two groups are shown in Table 1. Hemiparetic patients and the control group did not differ in regard to age, gender or anthropometric indices. Stroke survivors were worse educated, more often suffered from diabetes and less often suffered from osteoarthritis. Most of the patients underwent ischemic (62) and five hemorrhagic stroke.

**Table 1:** Participant characteristics

	Patients (*n* = 67)	Controls (*n* = 67)	*p* value
Age (years)	67.0 ± 9.2	66.9 ± 9.2	0.97
Height (m)	1.69 ± 0.08	1.70 ± 0.08	0.36
Body mass (kg)	77.8 ± 14.2	78.4 ± 14.8	0.72
BMI	27.3 ± 4.24	27.0 ± 3.41	0.86
Women; *n* (%)	22 (32.8)	22 (32.8)	1.0
Education			
Primary/vocational	30	7	<0.001
Secondary/higher	37	60	
Concomitant diseases			
Hypertension	45	37	0.21
Diabetes	22	7	0.003
Myocardial infarction	9	5	0.27
Osteoarthritis	12	29	0.002
Chronic pulmonary disease	6	6	1.00
Gastrointestinal disease	10	16	0.26

Muscle function characteristics (P_max_/kg, υ_opt_) as well as TUG results and deficits in comparison with control group are shown in Table 2. Significant deficits in P_max_/kg, υ_opt_ and TUG were observed in patients after stroke in comparison with participants from the control group both for men and women. Additionally, in post stroke patients there no sex differences in P_max_/kg, υ_opt_ or TUG results while in control group men had higher P_max_/kg and υ_opt_ than women.

**Table 2:** Comparison of P_max_, υ_opt_ and TUG results between post stroke patients and the control group

	Women				Men				All			
	Patients (*n* = 22)	Controls (*n* = 22)	% of controls	*p* value	Patients (*n* = 45)	Controls (*n* = 45)	% of controls	*p* value	Patients (*n* = 67)	Controls (*n* = 67)	% of controls	*p* value
P_max_/kg	2.25 ± 0.96	3.78 ± 1.61	59.5	<0.001	2.65 ± 1.56	5.72 ± 1.80	46.3	<0.001	2.52 ± 1.39	5.08 ± 1.96	49.6	<0.001
υ_opt_	47.3 ± 16.4	68.2 ± 17.4	69.4	<0.001	55.9 ± 19.5	87.3 ± 17.8	64.1	<0.001	53.1 ± 18.9	81.1 ± 19.8	65.5	<0.001
TUG	11.4 ± 7.9	7.6 ± 3.2	150.6	0.02	11.8 ± 7.2	6.8 ± 2.3	174.3	<0.001	11.7 ± 7.4	7.0 ± 2.6	166.0	<0.001

P_max_/kg and υ_opt_ were associated with TUG outcomes both in post stroke patients and control group participants (Table 3). Additionally, P_max_/kg and υ_opt_ were associated with age in the control group, but not in patients after stroke. TUG was more strongly related to age in healthy adults (controls) than in stroke patients (Table 3).

**Table 3:** Relationship between physical function measured with Timed Up and Go Test (TUG) and muscle function and age in post stroke patients and in the control group

	TUG	P_max_/kg	υ_opt_	Age
TUG		*ρ* = −0.53^a^ *p* < 0.001	*ρ* = −0.38^a^ *p* = 0.002	*ρ* = 0.60^a^ *p* < 0.001
P_max_/kg	*ρ* = −0.64^b^ *p* < 0.001		*ρ* = 0.72^a^ *p* < 0.001	*ρ* = −0.44^a^ *p* < 0.001
υ_opt_	*ρ* = −0.56^b^ *p* < 0.001	*ρ* = 0.81^b^ *p* < 0.001		*ρ* = −0.28^a^ *p* = 0.02
Age	*ρ* = 0.29^b^ *p* = 0.02	*ρ* = −0.21^b^ *p* = 0.09	*ρ* = −0.20^b^ *p* = 0.11	

a Controls.

b Patients.

In multivariate analysis with forward selection in patients after stroke, TUG was better predicted by P_max_/kg or υ_opt_ than by the age (Table 4). In contrast, in multivariate analysis in control group, TUG was better predicted by age than by P_max_/kg or υ_opt_ (Table 4).

Functional measurements specific for stroke patients. In functional measurements (not included in Table), the patients who had experienced a stroke obtained the following results: BI = 14.42 ± 4.33, RMA gross function = 8.51 ± 2.36, RMA arm function = 7.43 ± 4.14, RMA leg and trunk = 7.08 ± 2.39, and RMA total = 23.02 ± 7.48.

In stroke patients, both P_max_/kg and υ_opt_ were related to the BI (*ρ* = 0.48, *p* < 0.001 for P_max_/kg; *ρ* = 0.42, *p* < 0.001 for υ_opt_) and to the RMA total results (*ρ* = 0.58, *p* < 0.001 for P_max_/kg; *ρ* = 0.46, *p* < 0.001 for υ_opt_). Both BI and RMA total were not determined by age.

Further, multivariate analysis, including the results of P_max_/kg and age or υ_opt_ and age, was done to assess the most important determinants of BI and RMA total. For both BI and RMA total, age did not determine the variability of results.

For BI, P_max_/kg and υ_opt_ determined 23.73% and 17.17% of the variability of results, respectively.

For RMA total, P_max_/kg and υ_opt_ determined 32.63% and 22.69% of the variability of results, respectively.

**Table 4:** Multivariate analysis with forward selection for TUG test outcomes

Group	Analyzed determinants	Selected in the first step	Selected in the second step
Patients	P_max_/kg and age	P_max_/kg *R* ^2^ = 43.65%	Age *R* ^2^ = 47.35% (age – the remaining 3.7%)
	υ_opt_ and age	υ_opt_ *R* ^2^ = 30.76%	Age *R* ^2^ = 36.71% (age – the remaining 5.95%)
Controls	P_max_/kg and age	Age *R* ^2^ = 32.55%	P_max_/kg *R* ^2^ = 41.35% (P_max_/kg – the remaining 8.8%)
	υ_opt_ and age	Age *R* ^2^ = 32.55%	υ_opt_ *R* ^2^ = 40.95% (υ_opt_ – the remaining 8.4%)

## 4 DISCUSSION

Our study investigated the range of muscle function deficits and the relationship between muscle function and functional efficiency in stroke survivors in a relatively short period after the cerebrovascular incident.

### 4.1 Muscle function deficits in stroke survivors

We have shown that muscle power and muscle contraction velocity in patients who have suffered a stroke (up to three months after the incident) are significantly reduced as compared to healthy peers. The power generated by patients was only 49.6% that of the control group and muscle contraction velocity only 65.5%. In our preliminary study with power and velocity measured with the same methodology (Kostka, Fajkowska et al., 2017), we observed a slightly lower deficit for stroke patients (55.8% and 74.6% of the control group measurements for P_max_ and υ_opt_, respectively), but patients were more diverse in terms of time after stroke (13.68 ± 36.42 months). It has been reported that some degree of spontaneous recovery can be observed sometime after stroke onset, likely by reorganization of surviving central nervous system elements (Cramer, 2008). We have found no studies on muscle power deficits in patients in the same short period after stroke and only one that included patients in a period of 4–364 days after stroke (mean 70 ± 109 days) (Bohannon, 1992). In the aforementioned study, power and velocity (measured with a different methodology) were 42.7% and 70.7% in the unaffected side, respectively. It can be expected that deficits in comparison with healthy peers would be even higher because some of the authors indicate a deficiency in muscle strength or power in patients after stroke also in the unaffected side (Prado‐Medeiros et al., 2012; Stavric & McNair, 2012). For example, Stavric et al. (Stavric & McNair, 2012) reported that the knee extensor power of the control group was 35% higher than that of the affected limb of stroke survivors (at least 6 month after stroke).

In a few other articles on knee extensor muscles, power deficit after stroke was assessed in groups of patients in a later period after stroke than in our study, power measurements were made with a different methodology and separately for each leg (Hunnicutt & Gregory, 2017; Prado‐Medeiros et al., 2012; Saunders, Greig, Young, & Mead, 2008; Stavric & McNair, 2012). The method of measuring the muscular power used by us involved a specially prepared ergometer that allows not only for the measurement of muscle power value, but also reflects the ability of the limbs to cooperate, as is the case during basic everyday activities such as walking or climbing stairs. In most other studies, the deficit of muscle power in affected knee extensor muscles in relation to the unaffected side is between 43% and 65% (Hunnicutt & Gregory, 2017; Prado‐Medeiros et al., 2012; Stavric & McNair, 2012). However, in one study, it is only 10% (Saunders et al., 2008). This relatively small deficit found in that study probably results from the selection of participants: the study included patients who were ambulatory independent and had completed inpatient and outpatient stroke rehabilitation. This was explained by the authors by a good neurologic recovery (Saunders et al., 2008).

We wanted to indicate the actual deficits in muscle power and muscle shortening velocity resulting only from the stroke. In our study, results obtained by stroke survivors were compared with a group of people matched for age and sex who had not suffered a stroke. Participants from our two groups did also not differ in terms of anthropometric measurements like height, body mass and BMI. We tested our patients a relatively short time after stroke (<3 months). According to Jørgensen and Jacobsen (Jørgensen & Jacobsen, 2001), lean mass of both affected and unaffected limbs decreases greatly (5%–6%) within 2 months of a stroke. Nevertheless, we were not able to eliminate the pre stroke factors affecting muscle power and muscle contraction velocity. For example, it is known that active people are characterized by better muscle function (Rantanen, Era, & Heikkinen, 1997) and, at the same time, they are less likely to have a stroke (Lee, Folsom, & Blair, 2003). That is why it is possible that stroke survivors, even before the stroke, were characterized by lower muscle power.

### 4.2 Shortening velocity and functional performance

In our study, υ_opt_ was correlated with all of the functional measurements, both in the stroke patients and in the control group. This parameter for the knee extensor muscles may be measured during cycling, as was done in our study (Bonnefoy, Kostka, Arsac, Berthouze, & Lacour, 1998), or during knee extension exercises (Clémençon et al., 2008). The power‐velocity relationship drawn from this test allows for the calculation of the maximal power and velocity at which the power reaches a maximum value (optimal shortening velocity). This measure seems to be a very important indicator of functional performance. Clémençon et al. (2008) found that υ_opt_ was an even more important determinant of functional performance in elderly women than muscle power or muscle strength. Their results described 46% to 89% of the variance of functional tests used in that study (6‐m walking speed, chair‐stand time, and stair‐climb time) as predicted by υ_opt_. The relationship of velocity and everyday functioning was also observed by other authors, for example, in women with chronic osteoarthritis (Kostka et al., 2014) and in the elderly (Mayson, Kiely, LaRose, & Bean, 2008; Sayers, Guralnik, Thombs, & Fielding, 2005). Bohannon (Bohannon, 1992) analyzed maximal (not optimal) velocity in post stroke patients and found a relationship between maximal velocity and gait function.

### 4.3 Muscle power and functional performance

Other reports refer to the relationship between muscle power (measured with a different technique) and functional efficiency in stroke patients, particularly in regard to walking performance (Bohannon, 1992; Dawes et al., 2005; Saunders et al., 2008). In the study by Saunders et al (Saunders et al., 2008), lower limb extensor power in ambulatory people after stroke was a significant predictor of performance in functional tests. Participants with power lower than 1 W/kg were unable to get up from a chair without using their arms. Bohannon (Bohannon, 1992) reported a relationship between power of both paretic and nonparetic limbs with comfortable and maximum gait speed. Dawes et al. (2005) have shown that leg extensor power is related to walking performance after stroke (walking velocity, cadence, stance and swing time). In our study, P_max_/kg also significantly affected the results of all functional measurements. This relationship was clear, both for TUG, where P_max_/kg may directly influence an ability to perform activities like getting up from a chair and walking speed, but also in measurements that more generally evaluate functional performance, like IB and RMA.

### 4.4 Age versus muscle function and functional performance

In multivariate analysis of post stroke patients, as opposed to healthy controls, TUG was even better predicted by P_max_/kg or υ_opt_ than by the age. Similarly, both for BI and RMA, age did not determine the variability of stroke patients’ results. Interestingly, as opposed to results from our control group and other studies concerning elderly people, we obtained no relationship between either P_max_ or υ_opt_ and the age in stoke survivors. Therefore, the influence of the stroke, or rather the accumulation of deficits related to the stroke, has more harmful consequences for muscle performance than aging per se (Sions, Tyrell, Knarr, Jancosko, & Binder‐Macleod, 2012). In relatively healthy populations, gradual loss of muscle power and contraction velocity with age has been reported. Bonnefoy et al. (1998) indicated a 4.3% decline in υ_opt_ for every decade. This drop was already visible in people in their thirties and continued systematically until people reached their eighties (Kostka, 2005). The decrease in muscle power in the general population starts at about forty years of age and is more than 10% per decade (Kostka, 2005).

### 4.5 Practical applications

Many important independent functioning activities of daily living (rising from a sitting position, regaining balance after stumbling, walking, climbing and descending stairs, etc.) require the capacity to perform short, relatively intensive actions that demand the generation of appropriate muscle power. This has also been confirmed in the present study. The respondents who obtained worse P_max_ and υ_opt_ results were characterized by weaker results in functional tests.

Because of these consequences of aging for muscle power and, hence, for many ADLs, it is recommended to pay attention to muscle power in older population by utilizing resistance exercise training and by incorporating higher‐velocity training protocols (Chodzko‐Zajko et al., 2009). Due to the clear relationship between P_max_ and υ_opt_ in regard to functional performance of patients after a stroke, rehabilitation programs should include protocols that increase the power and velocity in this group of patients, also in the presence of concomitant diseases such as diabetes (Gray, Ivanova, & Garland, 2012; Orr, Tsang, Lam, Comino, & Singh, 2006). Such training seems to be safe and gives significant improvement in muscle power accompanied with functional gains (Hunnicutt et al., 2016; Morgan, Embry, Perry, Holthaus, & Gregory, 2015; Vinstrup, Calatayud, Jakobsen, Sundstrup, & Andersen, 2016). Because power is a product of force and velocity, various types of strength training (including high‐intensity training) can have a positive effect on muscle power and functional efficiency (Andersen et al., 2011; Vinstrup, Calatayud, Jakobsen, Sundstrup, Jay et al., 2016).

### 4.6 Study limitations

The method of measuring muscle function used in our study does not give the opportunity to evaluate the power separately for each leg. However, the advantage of such a measurement is the ability to show cooperation between the limbs, as it is the case during basic everyday activities (e.g., walking or climbing stairs).

The limits of the study are that the results come from a monocentric study and some selection bias cannot also be excluded.

## 5 CONCLUSIONS

Muscle power and muscle contraction velocity in patients who have had a stroke within three months are reduced markedly. These factors significantly affect functional performance. Muscle power and optimal shortening velocity are more important determinants of functional status than age in these stroke survivors. That is why rehabilitation programs for patients after stroke should include training that improves muscle power and incorporates higher‐velocity protocols.

## CONFLICT OF INTEREST

The authors declare that there are no conflicts of interest.
